# Knockdown of *β*-*N*-acetylglucosaminidase 2 Impairs Molting and Wing Development in *Lasioderma serricorne* (Fabricius)

**DOI:** 10.3390/insects10110396

**Published:** 2019-11-08

**Authors:** Wen-Jia Yang, Kang-Kang Xu, Xin Yan, Can Li

**Affiliations:** Guizhou Provincial Key Laboratory for Rare Animal and Economic Insect of the Mountainous Region, College of Biology and Environmental Engineering, Guiyang University, Guiyang 550005, China; yangwenjia10@126.com (W.-J.Y.); kkxu1988@163.com (K.-K.X.); 13885193419@163.com (X.Y.)

**Keywords:** *Lasioderma serricorne*, *β*-*N*-acetylglucosaminidase, molt, wing development, RNA interference

## Abstract

*β*-*N*-acetylglucosaminidases (NAGs) are carbohydrate enzymes that degrade chitin oligosaccharides into *N*-acetylglucosamine monomers. This process is important for chitin degradation during insect development and metamorphosis. We identified and evaluated a *β*-*N*-acetylglucosaminidase 2 gene (*LsNAG2*) from the cigarette beetle, *Lasioderma serricorne* (Fabricius). The full-length open reading frame of *LsNAG2* was 1776 bp and encoded a 591 amino acid protein. The glycoside hydrolase family 20 (GH20) catalytic domain and an additional GH20b domain of the LsNAG2 protein were highly conserved. Phylogenetic analysis revealed that LsNAG2 clustered with the group II NAGs. Quantitative real-time PCR analyses showed that *LsNAG2* was expressed in all developmental stages and was most highly expressed in the late larval and late pupal stages. In the larval stage, *LsNAG2* was predominantly expressed in the integument. Knockdown of *LsNAG2* in fifth instar larvae disrupted larval–pupal molting and reduced the expression of four chitin synthesis genes (trehalase 1 (*LsTRE1*), UDP-*N*-acetylglucosamine pyrophosphorylase 1 and 2 (*LsUAP1* and *LsUAP2*), and chitin synthase 1 (*LsCHS1*)). In late pupae, *LsNAG2* depletion resulted in abnormal adult eclosion and wing deformities. The expression of five wing development-related genes (teashirt (*LsTSH*), vestigial (*LsVG*), wingless (*LsWG*), ventral veins lacking (*LsVVL*), and distal-less (*LsDLL*)) significantly declined in the *LsNAG2*-depleted beetles. These findings suggest that *LsNAG2* is important for successful molting and wing development of *L. serricorne*.

## 1. Introduction

Chitin is a polymer of *β*-1,4-linked *N*-acetylglucosamine residues and an essential component of the integument, trachea, salivary gland, foregut, hindgut, and intestinal peritrophic matrix in insects [1,2]. Chitin plays major roles in maintenance of the shape, size, and protection from external forces such as mechanical injuries and infection by bacteria, fungi, and viruses [3,4]. The regulation of chitin metabolism is crucial for insect growth and development [5,6]. Among the enzymes involved in chitin metabolism, *β*-*N*-acetylglucosaminidases (NAGs, EC 3.2.1.30) are chitin-degrading enzymes that hydrolyze the chitin oligosaccharides into *N*-acetylglucosamine monomers [7]. NAGs (also known as *β*-*N*-acetylhexosaminidases) are exosplitting enzymes that belong to the glycoside hydrolase family 20 (GH20) [8]. Insect NAGs are divided into four subgroups: chitinolytic group I NAGs, chitinolytic group II NAGs, glycan processing group III NAGs, and hexosaminidases group IV, on the basis of their sequence similarity and functions [9,10,11].

Insect NAGs are widely distributed and occur in many insect species, including Coleoptera, Diptera, Hemiptera, Lepidoptera, and Orthoptera [10,11,12,13,14,15]. NAGs mainly function in chitin degradation, and their roles have been studied in numerous insect species. In *Aedes aegypti*, the activity of NAG enzyme increased after feeding on blood or artificial diet [16]. RNA interference (RNAi)-mediated knockdown of *NAGs* causes molting failure in *Tribolium castaneum* [10], *Locusta migratoria* [13], *Nilaparvata lugens* [11], *Mamestra brassicae* [14], and *Heortia vitessoides* [17]. In *Ostrinia furnacalis* [17] and *Lasioderma serricorne* [18], *NAGs* are essential for the proper formation of adult wings. In addition to their central role in chitin degradation, NAGs participate in diverse physiological processes, such as post-translational modification of *N*–glycan proteins [19], degradation of glycoconjugates [20,21], and fertilization [22,23].

The cigarette beetle, *Lasioderma serricorne* (Fabricius) (Coleoptera: Anobiidae), is a serious pest of stored products worldwide [24]. The beetle causes economic losses to stored products such as tobacco, grains, traditional Chinese medicine materials, cereals, and dry foods [25,26]. Control of *L. serricorne* is primarily dependent on the use of insecticides, including phosphine, essential oils, pyrethrin, and organophosphates [27,28]. Excessive and frequent insecticide applications have resulted in insecticide resistance within many *L. serricorne* populations. In addition, insecticide residues in stored products are harmful to human health and the environment [29,30]. Therefore, new strategies are needed to effectively manage *L. serricorne* infestations. RNAi-based technologies have been used for the management of several insect pests [31,32,33]. Screening for an ideal silencing target is critical for the development of an RNAi-based control method [34,35]. In terms of practice, transgenic plants in expressing sequence-specific double-stranded RNA (dsRNA) [36] and spray-based dsRNA reagent application [37] have potential in implementation as future pest control, and a trial of *Diabrotica virgifera virgifera* via targeting DvSnf7 was firstly tested in filed as RNAi-based pest control incoperated with *Bacillus thuringiensis* Cry3Bb1 protein [38]. Chitin degrading enzymes are potential targets for novel insecticides [17,39,40,41]. In this study, we identified and cloned the full-length open reading frame (ORF) sequence of *β*-*N*-acetylglucosaminidase 2 gene (*LsNAG2*) in *L. serricorne* and analyzed its expression patterns in different developmental stages and tissues. We functionally characterized the knockdown effect of *LsNAG2* on *L. serricorne* molting and the responses of genes related to wing development. The data provide significant insight into the functions of *LsNAG2* in *L. serricorne* development.

## 2. Materials and Methods

### 2.1. Insect Culture

*Lasioderna serricorne* were collected from a tobacco warehouse in Guiyang City, Guizhou Province, China, in 2014. A colony was established in the laboratory and maintained at 28 °C with 40% relative humidity under a scotoperiod of 24 h. Larvae were fed on dried roots of *Angelica sinensis*, as described previously [42].

### 2.2. RNA Extraction and Cloning of LsNAG2 Gene

Total RNA was extracted from entire bodies of 30 *L. serricorne* larvae with TRIzol reagent according to manufacturer protocol (Invitrogen, Carlsbad, CA, USA), and treated with DNase I (Promega, Madison, WI, USA). The concentration and quantity of total RNA were measured by a NanoDrop 2000C spectrophotometer (Thermo Scientific, Waltham, MA, USA). RNA integrity was evaluated by agarose gel electrophoresis. First-strand complementary DNA was synthesized by the TransScript Synthesis Supermix (TransGen, Beijing, China) with oligo (dT)_18_ primers.

A candidate cDNA sequence of *β*-*N*-acetylglucosaminidase 2 gene was identified by Basic Local Alignment Search Tool Nucleotide (BLASTN) and Translated Basic Local Alignment Search Tool Nucleotide (TBLASTN) analyses from the transcriptome database of *L. serricorne* (unpublished data). The full-length ORF of the sequence was found using ORF Finder at the National Center for Biotechnology Information (NCBI) website (http://www.ncbi.nlm.nih.gov/gorf/gorf.html). Two pairs of gene-specific primers (Table S1) were designed to amplify the full-length cDNA of *LsNAG2* by reverse transcription PCR (RT-PCR). The PCR amplification was carried out with an initial denaturation at 95 °C for 4 min, 34 cycles of 95 °C for 30 s, 56 °C for 30 s, and 72 °C for 2 min, and a final elongation at 72 °C for 10 min. The PCR products were purified using a Wizard SV Gel and PCR Clean-Up System (Promega, Madison, WI, USA) and sequenced in both directions.

### 2.3. Sequence Analysis

Sequence similarities and domain predictions were produced using the Basic Local Alignment Search Tool program at the NCBI website (http://blast.ncbi.nlm.nih.gov/). The nucleotide and deduced amino acid sequences were analyzed using DNAMAN7 (Lynnon Biosoft, Vaudreuil, Quebec, Canada). The molecular weight and isoelectric point were calculated using ExPASy tools (http://cn.expasy.org/tools/pi_tool.html). The *N*-glycosylation sites were predicted using the NetNGlyc1.0 Server (http://www.cbs.dtu.dk/services/NetNGlyc/), and the signal peptide was predicted using the SignalP4.1 program (http://www.cbs.dtu.dk/services/SignalP4.1/). The phylogenetic tree was constructed by MEGA7 (MEGA, PA, USA) [43] using the neighbor-joining method with bootstrap values using 1000 replicates at the cut off of 50% similarity.

### 2.4. Quantitative Real-Time PCR

For the stage-specific expression analyses, samples at different developmental stages of *L. serricorne* were prepared as described previously [42]. To analyze tissue-specific expression, eight tissues (integument, brain, foregut, midgut, hindgut, fat body, and Malpighian tubules) were dissected from the late larvae. RNA extraction and cDNA synthesis were performed as described above. The relative expression levels of *LsNAG2* were detected by quantitative real-time PCR (qPCR). The qPCR assay was performed with GoTaq qPCR Master Mix (Promega, Madison, WI, USA) using the CFX-96 real-time PCR system (Bio-Rad, Hercules, CA, USA). The reaction was run at 95 °C for 5 min, followed by 40 cycles of 95 °C for 15 s and 60 °C for 1 min. A melt curve analysis was performed to assess the specificity of the qPCR amplifications. On the basis of the evaluation, the *L. serricorne* elongation factor 1-alpha (EF1α, GenBank: KY549658) was used for normalization (unpublished data). All of the experiments were performed in triplicate, and the results were calculated by the 2−ΔΔCt method [44].

### 2.5. RNA Interference

RNAi was performed to study the effects of *LsNAG2* on *L. serricorne* development. The primers (Table S1) used for dsRNA synthesis were designed to add the T7 polymerase promoter sequence at the 5′-ends. The dsRNAs were synthesized using a TranscriptAid T7 High Yield Transcription Kit (Thermo Scientific, Wilmington, DE, USA). The quality and concentration of dsRNAs were measured with a NanoDrop 2000C spectrophotometer (Thermo Scientific), and their sizes were verified by agarose gel electrophoresis. The dsRNA of green fluorescent protein (*GFP*) served as the negative control. The day 2 fifth-instar larvae and day 5 pupae were used for microinjection; 50 individual insects were used for each group, and the experiment was performed in triplicate. About 300 ng dsRNA of *GFP* or *LsNAG2* were slowly injected into the hemocoel between the second and third abdominal segments of each larva and pupa using a Nanoliter 2010 injector (World Precision Instruments, Sarasota, FL, USA). All of the injected insects were kept under the conditions mentioned above. To determine silencing efficiency, relative expression levels of *LsNAG2* in both ds*LsNAG2* and ds*GFP* group at 3 and 5 days after injection were measured by qPCR as described above. Insect survival rate and phenotype changes were observed and recorded. Photos were taken using a Keyence VHX-6000 stereomicroscope (Keyence Corporation, Osaka, Japan).

To determine the expression of chitin synthesis genes, samples were collected from the larvae treated with ds*LsNAG2* and ds*GFP* for 5 days. The qPCR was used to analyze the expression of trehalase (*LsTRE1* and *LsTRE2*), UDP-*N*-acetylglucosamine pyrophosphorylase (*LsUAP1* and *LsUAP2*), and chitin synthase 1 (*LsCHS1*) as described above. We also evaluated the expressions of wing development-related genes after gene silencing in late pupae. The insect samples were collected 5 days after dsRNA injection in late pupae for expression analysis. The relative expression levels of nine wing development network genes, including miniature (*LsMT*), singed (*LsSG*), engrailed (*LsEN*), teashirt (*LsTSH*), wingless (*LsWG*), distal-less (*LsDLL*), vestigial (*LsVG*), ventral veins lacking (*LsVVL*), and apterous (*LsAP*), were detected by qPCR.

### 2.6. Statistical Analysis

All the expression data are presented as mean ± standard error (SE) and evaluated using SPSS 20.0 software (IBM Corp, Chicago, IL, USA). One-way analysis of variance (ANOVA) followed by a least significant difference test was used to analyze differences among different developmental stages and tissues. Student’s *t*-test was used to analyze data from the RNAi-treatments after dsRNA injection. The survival rates were analyzed using the Kaplan–Meier method.

## 3. Results

### 3.1. Identification and Characterization of LsNAG2

The full-length ORF of *LsNAG2* (GenBank accession number MN310607) was obtained and confirmed by RT-PCR. The *LsNAG2* cDNA sequence contained an ORF of 1776 bp that encoded a protein of 591 amino acids. The predicted protein of *LsNAG2* had a molecular weight of 66.6 kDa and an isoelectric point of 5.92. Signal peptides with 16 amino acids were found at the N-terminal ends of LsNAG2, which suggested it may be secreted. Two potential *N*-glycosylation sites at positions 150 and 549 were predicted in the N-terminal extracellular domain.

**Figure 1:**
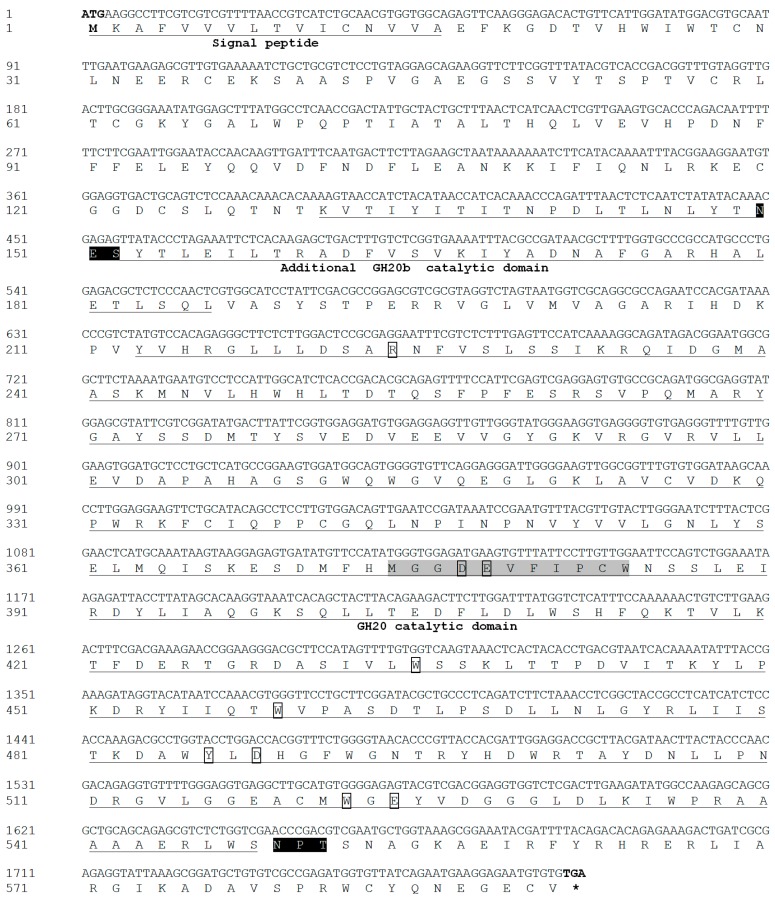
Nucleotide and deduced amino acid sequences of *LsNAG2* complementary DNA. The start codon is indicated in bold and the stop codon in bold with an asterisk. The predicted signal peptide, glycoside hydrolase family 20 (GH20) catalytic domain, and an additional GH20b domain are underlined. The conserved catalytic sites are shaded. The potential glycosylation sites are in white with a blank background, and the catalytically active sites are boxed.

**Figure 2:**
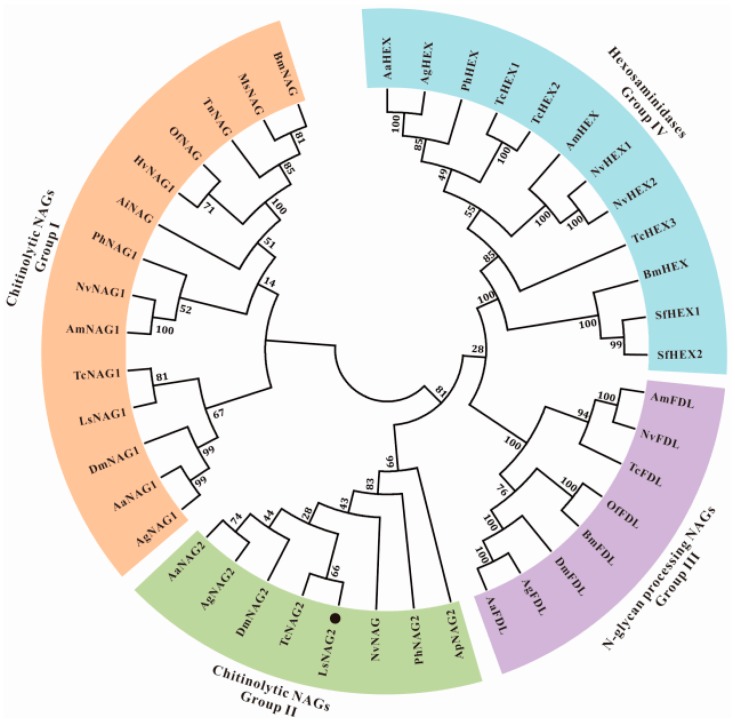
Phylogenetic analysis of insect *β*-*N*-acetylglucosaminidases. The phylogenetic tree was constructed using neighbor-joining method and bootstrap support values on 1000 replicates by MEGA7. LsNAG2 is marked with black dots. Table S2 shows the protein sequences of the *β*-*N*-acetylglucosaminidases used in the phylogenetic tree.

The predicted amino acid sequences contained a conserved GH20 catalytic domain (residues 213–572), and an additional GH20b domain (residues 136–186). The conserved H×GGDEV×××CW motif was considered to be the catalytic site. The conserved active site residues R224, D377, E378, W435, W459, Y486, D488, W522, and E524 were found in the LsNAG2 sequence (Figure 1). Sequence alignment showed that LsNAG2 exhibited 60%, 54%, 53%, and 52% identity with NAG2 (XP_018336054) from *Agrilus planipennis*, NAG2 (XP_015833058) from *T. castaneum*, NAG (XP_023711735) from *Cryptotermes secundus*, and NAG (XP_022185539) from *Nilaparvata lugens*, respectively. A phylogenetic tree was generated using MEGA7 on the basis of the amino acid sequences of insect NAGs and related hexosaminidases. The tree showed that these proteins were classified into four major groups: NAG group I, NAG group II, *N*-glycan processing NAGs (group III), and hexosaminidase (group IV). The LsNAG2 protein was located on a branch with the group II NAGs of other insects (Figure 2).

### 3.2. Spatiotemporal Expression Patterns of LsNAG2

**Figure 3:**
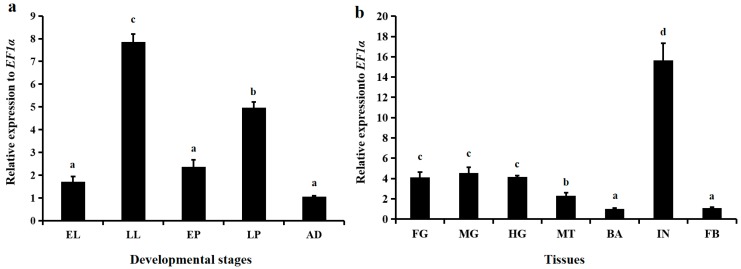
The spatiotemporal expression patterns of *LsNAG2* in *Lasioderma serricorne*. (**a**) The relative expression levels of *LsNAG2* in different developmental stages. EL, early larave; LL, late larvae; EP, early pupae; LP, late pupae; AD, adults. (**b**) The relative expression levels of *LsNAG2* in different tissues including integument (IN), brain (BA), fat body (FB), foregut (FG), midgut (MG), hindgut (HG), and Malpighian tubules (MT). Elongation factor 1-alpha (EF1α) was used as the reference control. Lowercase letters above bars indicate statistical difference based on one-way ANOVA followed by a least significant difference test (*p* < 0.05).

Among the five tested stages, that is, early larvae (<24 h post-hatching), late larvae (older than fourth instar but before prepupae), early pupae (>72 h post-pupation), late pupae (>144 h post-pupation), and adults (>72 h post-eclosion), *LsNAG2* was continuously expressed but displayed different expression patterns. *LsNAG2* was more highly expressed in the late larval and late pupal stages than in the other stages (Figure 3a). The late larval stage had the highest expression of *LsNAG2* and was investigated for spatial expression patterns. The highest expression level of *LsNAG2* was observed in the integument and was approximately 15.6-fold greater than in the brain (Figure 3b).

### 3.3. Effects of LsNAG2 RNAi on the Expressions of Chitin Synthesis Genes

**Figure 4:**
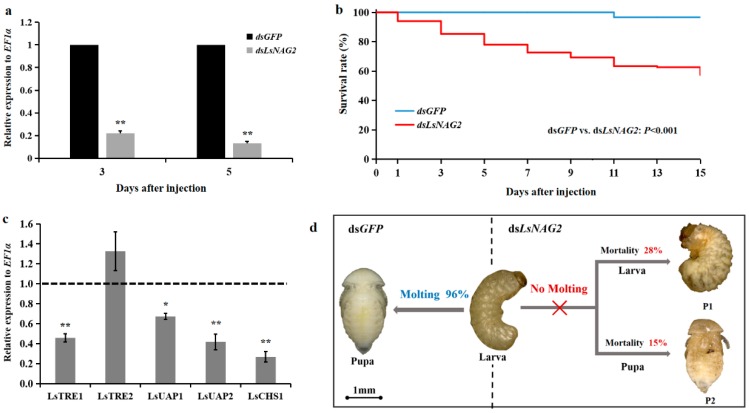
Effect of *LsNAG2* RNA interference (RNAi) on the larval–pupal transition in *L. serricorne*. (**a**) Relative expression levels of *LsNAG2* at 3 and 5 days after the *LsNAG2* or green fluorescent protein (*GFP*) double-stranded RNA injection at day 2 of fifth-instar larvae. (**b**) Changes in survival of *L. serricorne* larvae after *LsNAG2* or *GFP* dsRNA injection. (**c**) Effects of *LsNAG2* knockdown on the expression of chitin synthesis pathway genes. The expression level of chitin synthesis pathway genes in the control is marked with a dashed line. (**d**) Representative phenotypes of the larvae after *LsNAG2* or *GFP* dsRNA injection. Injection of ds*LsNAG2* resulted in two lethal phenotypes. P1: Larvae were unable to molt, retained the larval form, and ultimately died; P2: insects shed their old cuticles and completed the larval–pupal transformation but died after molting. Larvae were alive and successfully molted to pupae at day 5 after injection with ds*GFP*. Significant differences between treatment and control with Student’s *t*-test are indicted by * *p* < 0.05, ** *p* < 0.01.

To study the functions of *LsNAG2* in the molting process, specific dsRNA for *LsNAG2* and *GFP* were synthesized in vitro and injected into fifth instar larvae and late pupae. Compared to the control, the expression of *LsNAG2* was significantly reduced by 78% and 87% at 3 and 5 days after injection with ds*LsNAG2* in the fifth instar larvae, respectively (Figure 4a). After inhibition of *LsNAG2*, the survival rate of *L. serricorne* was reduced to 57% at 15 days in the ds*LsNAG2* group (Figure 4b).

After RNAi with ds*LsNAG2* or ds*GFP* at larvae, qPCR was used to study the expression of chitin synthesis genes. Expression levels of *LsTRE1*, *LsUAP1*, *LsUAP2* and *LsCHS1* were significantly decreased in larvae treated with ds*LsNAG2* compared to levels in control insects (Figure 4c). This suggested that *LsNAG2* is involved in the regulation of genes involved in chitin synthesis during the larval–pupal transition. The lethal phenotypes included the following: (1) 28% of the individuals were unable to molt, retained the larval form, and ultimately died; and (2) 15% of the individuals eventually shed their old cuticles and completed the larval–pupal transformation but died after molting (Figure 4d).

### 3.4. Effects of LsNAG2 RNAi on the Expressions of Wing Development-Related Genes

**Figure 5:**
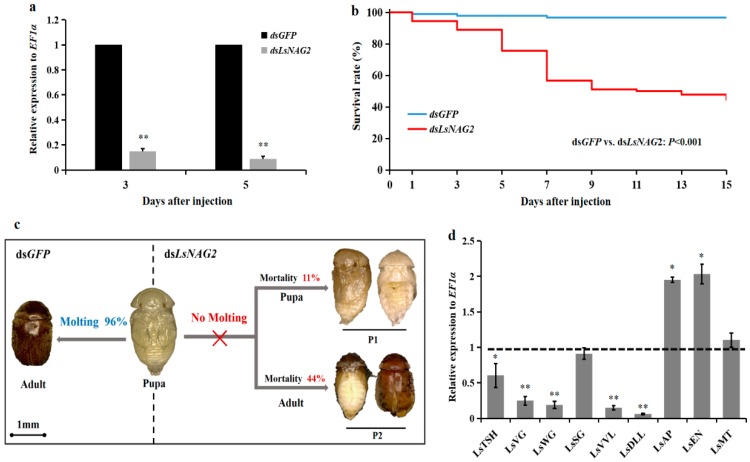
Effect of *LsNAG2* RNAi on the pupal–adult transition in *L. serricorne*. (**a**) Relative expression levels of *LsNAG2* at 3 and 5 days after the *LsNAG2* or *GFP* dsRNA injection at day 5 of pupae. (**b**) Changes in survival of *L. serricorne* pupae after *LsNAG2* or *GFP* dsRNA injection. (**c**) Representative phenotypes of the pupae after *LsNAG2* or *GFP* dsRNA injection. Injection of ds*LsNAG2* resulted in two lethal phenotypes. P1: pupae were unable to shed off their old cuticles and died without completing adult eclosion; P2: pupae molted to adults but died with an asymmetric wing pattern. Pupae were alive and successfully molted to adults at day 3 after injection with ds*GFP*. (**d**) Effects of *LsNAG2* knockdown on the expression of genes involved in wing development. The expression levels of wing development-related genes in the control is marked with a dashed line. Significant differences between treatment and control with Student’s *t*-test are indicted by * *p* < 0.05, ** *p* < 0.01.

Injection of late pupae with ds*LsNAG2* significantly decreased the expression of *LsNAG2* by 85% and 91% at 3 and 5 days, respectively, compared to the control (Figure 5a). In the control group, 96% of pupae molted normally to adults during the pupa to adult transition. Pupae injected with ds*LsNAG2* exhibited lethal phenotypes. The accumulative survival rate of pupae was reduced to 45% at 15 days after treatment compared with the controls (Figure 5b). Approximately 11% of the individuals had difficulty shedding the old cuticle, failed to develop into adults, and eventually died. Interestingly, 44% of the individuals successfully molted but they did not complete wing development and died with an asymmetric wing pattern (Figure 5c).

To detect effects of ds*LsNAG2* injection on wing development, we examined the expression of nine genes related to wing development after RNAi with ds*LsNAG2* or ds*GFP* at late pupae. The qPCR results showed that the mRNA levels of five transcripts (*LsTSH*, *LsVG*, *LsWG*, *LsVVL*, and *LsDLL*) were significantly reduced in the *LsNAG2*-depleted pupae, compared to levels in the controls (Figure 5d). In contrast, the expression levels of *LsA*P and *LsEN* were significantly higher in *LsNAG2* RNAi pupae.

## 4. Discussion

Chitin synthesis and chitin degradation are both important in insect development [5]. Chitinases and NAGs are critical enzymes for insect chitin metabolism. Chitinases hydrolyze chitin into oligosaccharides, whereas NAGs further degrade the small oligomers into *N*-acetylglucosamine monomers [7]. Several *NAG* genes have been characterized in many insect species. There are 4 in *T. castaneum* [10], 6 in *Bombyx mori* [45], 5 in *Drosophila melanogaster* [22], and 11 in *N. lugens* [11]. In *L. serricorne*, one *NAG* gene (*LsNAG1*) has been previously characterized [18]. In this study, we describe a second *NAG*, *LsNAG2*, in the cigarette beetle *L. serricorne*. The GH20 and GH20b domains were predicted in LsNAG1 and LsNAG2 proteins, which indicated that these two genes belong to the glycoside hydrolase family 20. Both domains were also predicted in the NAG of *Exopalaemon carinicauda* [46]. The phylogenetic relationships indicate that LsNAG2 belongs to the chitinolytic group II NAG [10]. All insect NAGs are related to chitin degradation, but group I and II are well characterized in chitin decomposition. The sequence characterization and phylogenies showed that TcNAG2 in *T. castaneum* and DmNAG2 in *D. melanogaster* are closely related and LsNAG2 possibly has a chitin degradation function [10,20].

*NAGs* are widely expressed in all developmental stages of insects because of their multiple physiological functions. For example, four *NAGs* are expressed in all stages of *N. lugens* [11]. In this study, *LsNAG2* was expressed in all tested stages of *L. serricorne* but most highly expressed in late instar larvae and late pupae. This indicates its vital role in the degradation of old cuticle during molting. Similar high expression in late larvae and late pupae also occur in *LsNAG1* [18]. High expression of *NAG* was observed at late fourth and fifth instar larvae of *H. vitessoides* [16] and *O. furnacalis* [47]. High expression was also seen in the last instar larvae of *M. brassicae* [14]. Increased expression of *TcNAG1* and *TcNAG3* was investigated in late instar larvae and pupae of *T. castaneum*, respectively [10]. Several *NAGs* were also highly expressed in the early pupal stage in other insects [10,14].

Insect *NAGs* are widely expressed in multiple tissues [11,45]. In *N. lugens*, a group II *NAG* (also defined as *β*-*N*-acetylhexosaminidases) gene, *NlHex3*, was stably expressed in all tissues, whereas the others showed a tissue-biased pattern, such as the gut, Malpighian tubules, salivary gland, and reproductive organ [11]. The spatial expression indicated tissue-specific functions of NAGs in insects. In *B. mori*, ovary- and testis-specific expressions were observed [45]. A fertilization-related function was demonstrated in *D. melanogaster*, in which three *NAG* genes were expressed in the male germ line [22]. In this study, *LsNAG2* was prominently expressed in integument. A similar expression pattern was also demonstrated in *NlHex4* of *N. lugens* [11]. The high expression of *NAGs* in integument may be associated with chitin metabolism during the molting process. In *Choristoneura fumiferana*, an immunohistochemical staining assay revealed that *CfNAG* occurred in the degrading exocuticle and molting fluid during late ecdysis [48]. Ecdysone is a hormone involved in the insect molting process, and it helps to regulate NAG activity during development. In *H. vitessoides* and *M. brassicae*, the *NAG* gene was upregulated by exogenous 20-hydroxyecdysone [14,16]. This induced expression can be suppressed by juvenile hormone [7]. The 20E-induced expression was studied in *LsNAG1* and shown to be involved in the larval–pupal transition [18].

The suppressed *LsNAG1* disrupted the metamorphic transition from larva to pupa and pupa to adult [18]. The expression profiling also showed a similar role in the transition. When the *LsNAG2* was successfully suppressed by exogenous dsRNA micro-injection, almost 43% of the larvae exhibited a lethal phenotype (failure to successfully molt). A similar disruption was observed in *N. lugens* when *NlHex4* was suppressed, but it did not reveal any abnormal phenotypes in the remaining 10 NAG homologs [11]. In *L. serricorne*, suppression of *LsNAG2* led to molting failure of *LsNAG1* and chitin deacetylase 1 gene [18,49]. The function of *NAGs* in molting was also studied in *L. migratoria* [13], *M. brassicae* [14], and *O. furnacalis* [18,47]. NAGs were present in the molting fluid and showed a positive synergistic effect on chitin degradation together with chitinases [50,51]. Gene expression of the chitin synthesis pathway was studied by qPCR, and four out of the selected five genes were downregulated by silencing *LsNAG2*. This indicated that the disrupted chitin degradation also inhibited new chitin biosynthesis when the chitin metabolism-related *LsNAG2* was suppressed at the transcriptional level. However, the relative expression of the chitin synthesis-related genes was not influenced when *NAG* was knockdown by dsRNA in *H. vitessoides* [16]. In addition, the genes involved with chitin degradation showed no change when *HvNAG1* was silenced. Group I NAGs are all secreted proteins that have been isolated from molting fluid, integument, and gut. They are functionally responsible for chitin degradation in the cuticle [51]. In this study, *LsNAG2* had a role similar to other group I NAGs and was involved in chitin degradation within the cuticle. The phenotypes produced in *T. castaneum* by knocking down the other NAGs was studied, and the efficiency was not as strong as that produced by the group I NAG (*TcNAG1*) [10].

In *M. brassica*, suppression of *NAG* expression in larvae also increased mortality at the pupal-adult transition [14]. The continuous suppression was also reflected in the following stages [13,16]. We studied the role of *LsNAG2* in the pupa-adult transition and observed high mortality when *LsNAG2* was silenced. The higher mortality of *LsNAG1* indicated a stringent efficiency during pupal–adult molting, which can also be concluded by the comparison of accumulative mortality of *NAG1* and *NAG2* knocked phenotype in *L. serricorne* [10,18]. Similar to the *LsNAG1* RNAi bioassay, asymmetric wing development was also observed in the *LsNAG2* RNAi, indicating a critical role in the wing development. Suppressed *HvNAG1* in *H. vitessoides* also led to an asymmetric wing pattern in adults [16]. In *O. furnacalis*, suppressed *OfHex2* resulted in 23.3% of the adults having abnormal wings [17]. The relationship of chitin-degrading enzymes and the upstream chitinase 7 with wing development was also documented in *T. castaneum* [52]. However, the mechanism of this abnormality in wing development is unclear. Therefore, we studied the gene expression of several wing development-related genes after RNAi of *LsNAG2*. Five out of the nine candidate genes showed downregulated expression. These genes are involved in wing development. In *N. lugens*, downregulated expression of *apterous* and *teashirt* genes induced by the suppressed *trehalase* gene resulted in wing deformity [53]. The vital roles of wingless and vestigial involved in wing development have been studied in *Drosophila* [54]. In *T. castaneum*, RNAi analysis showed that the vestigial gene is essential for normal wing formation [55]. Downregulation of such genes indicates that chitin metabolism may be functionally related with wing development and formation. Given that NAG is crucial for insect molting and wing development, it may be possible to serve as a candidate insecticide target. Further studies are needed for developing transgenic plants expressing specific NAG dsRNA or spray-based dsRNA reagents to control insect pests.

## 5. Conclusions

We identified and cloned a *NAG* gene (*LsNAG2*) in *L. serricorne*, which belongs to the group II NAG. Quantitative expression analysis showed that *LsNAG2* was predominantly expressed during the larval–pupal and pupal–adult molting transitions, especially in the integument tissue. An RNAi bioassay showed that *LsNAG2* is functionally involved in metamorphic transitions by regulating the chitin degradation of the old cuticle. *LsNAG2* was also involved in the wing development of *L. serricorne*. These results demonstrated that *LsNAG2* is a promising RNAi target for *L. serricorne* control, and this gene might be useful for developing novel pest control strategies.

## Supplementary Materials

The following are available online at https://www.mdpi.com/2075-4450/10/11/396/s1: Table S1: Primers used in this study. Table S2: Details of *β*-*N*-acetylglucosaminidase protein sequences used for phylogenetic analysis.
